# Awareness toward stroke in a population-based sample of Iranian adults

**Published:** 2017-01-05

**Authors:** Mozaffar Hosseininezhad, Hannan Ebrahimi, Seyed Mohammad Seyedsaadat, Babak Bakhshayesh, Motahareh Asadi, Amir Reza Ghayeghran

**Affiliations:** 1 Department of Neurology, Poursina Hospital, Guilan University of Medical Sciences, Rasht, Iran; 2 Student Research Committee, School of Medicine, Guilan University of Medical Sciences, Rasht, Iran; 3 Department of Neurology, School of Medicine, Poursina Hospital, Guilan University of Medical Sciences, Rasht, Iran

**Keywords:** Awareness, Stroke, Signs and Symptoms, Risk Factors

## Abstract

**Background:** Stroke is the leading cause of death and functional disability. While there have been major advances regarding the management of stroke, a significant proportion of people are still unaware of stroke-related symptoms and risk factors. This study was performed to assess the awareness of stroke’s warning signs and risk factors among a sample of Iranian population.

**Methods:** A total of 649 participants were randomly selected using systematic randomization from the list of telephone numbers obtained from the telephone directory. Demographic characteristics were recorded. Participants were asked to answer questions regarding the awareness about stroke, its warning signs and risk factors.

**Results:** Patients’ mean age was 32.0 ± 12.2 years old, and 56.4% were women. Hypertension and history of stroke were major risk factors, and loss of consciousness, vertigo and ataxia were major warning signs of stroke correctly identified by respondents. Multiple linear regressions showed that age (β = 0.277, P < 0.001), academic level of education (β = 6.41, P = 0.01), housewifery (β = 8.9, P < 0.001), jobs related to medical care (β = 13.17, P = 0.016) and previous information about stroke (β = 18.71, P < 0.001) were significant predictors of the overall awareness about stroke.

**Conclusion:** The awareness of people about stroke, its risk factors and warning signs were good in this study. The awareness toward stroke can be associated with factors such as age, academic level of education, job and previous information about stroke. Further studies are recommended to program public multimedia and health education in academies and colleges.

## Introduction

Stroke is the second cause of death and the leading cause of long-term disability worldwide.^1^^-^^3^ While there has been a decrease in the incidence of stroke over the past forty years in western countries, the incidence of stroke in the same time period has increased in developing countries.^1^^,^^4^ Recent study showed a considerably higher incidence of stroke among Iranian population than most other regions of the world.^5^ A number of reasons have been suggested to explain the higher incidence and mortality rate in low to middle-income countries, including population growth, aging, adoption of a sedentary lifestyle and poor dietary habits, increased disease-related risk factors and lack of public knowledge about stroke.^6^^-^^8^

Lack of knowledge regarding main clinical presentations of the disease is a major health problem which leads to prolonged time elapsed from the onset of stroke to hospitalization, late diagnosis and therefore delayed start of appropriate treatment. The knowledge and awareness of people about symptoms, warning signs and risk factors of stroke is crucial to prevent stroke by reducing the number of patients who are at a higher risk of the disease and to help patients to seek immediate medical care and receive timely diagnosis and life-saving treatment by the rapid detection of those at a higher risk of developing neurovascular events.^8^^,^^9^

While there have been major advances toward the management of stroke since few years ago, significant proportion of people are still unaware of stroke-related symptoms and risk factors.^10^^,^^11^ In addition to developing countries,^7^^,^^8^^,^^10^^,^^12^^,^^13^ in many developed nations like Canada,^14^ the USA,^15^^-^^19^ South Korea,^20^ Australia,^21^ France,^22^ and Denmark^11^ lots of stroke patients are not presented to the emergency department in timely manner to receive appropriate treatment due to their inadequate knowledge about major warning signs and risk factors of the disease.^10^ Cossi, et al.^8^ demonstrated that more than 33.0% of participants were able to recognize at least one stroke symptom, and more than 55.2% were aware of stroke risk factors. They found that paralysis or hemiplegia was the most frequent symptom identified by 34.4%, and hypertension was the major risk factor identified by 34.5% of individuals. 

Although abundant studies have been conducted to survey the public knowledge of stroke in western nations, far too little attention has been paid to this important issue in developing countries, especially in Iran where the average age of population, epidemiologic features and risk factors of stroke, availability of information, education, training, and medical care facilities are different. In addition, considering the marked increase in the incidence of stroke in developing countries, especially in Iran,^4^^,^^5^ findings of such study are of great interest that help us to know the knowledge of our population about stroke and understand the extent of the problem. This will help us to adopt more effective, comprehensive, educational programs to increase the public knowledge of stroke and therefore reduce the burden of stroke. This study was therefore conducted to evaluate the public awareness regarding risk factors and warning signs of stroke among a sample of Iranian population. To the best of our knowledge, this study is the first population-based survey that assesses the level and the factors related to stroke awareness among Iranian population.

## Materials and Methods

Guilan province is located in the northern part of Iran and forms the southwest border of the Caspian Sea. The province extends over 14000 km^2^ and has inhabitants of about 2.5 million people. Rasht, the capital of Guilan, is the most populous and the largest city along the Caspian Sea coast. 

This cross-sectional, population-based telephone survey was carried out between May and July 2012 in Rasht, Iran. The study was approved by the ethic and faculty research committee of the Guilan University of Medical Sciences (GUMS). A total of 649 households were randomly selected using a systematic randomization from the list of telephone numbers obtained from the information service of the telephone directory and then contacted by telephone call. To avoid probable selection bias, the participants were randomly selected from the three socioeconomically different districts of the city. Individuals who were 15 years or more and consented to participate in our research were initially included in the study. The other phone number was substituted when the eligible person was not available on the first phone call to answer our questions. The interviewers first introduced themselves and briefly explained the aim of the study to respondents and then asked them if they were interested to participate in this research. Two medical interns of GUMS were trained and given instructions to implement a telephone interview and clarify any ambiguous question if needed. Respondents’ answers were recorded without the direct intervention of interviewers. All questions were closed-ended and were asked in Persian.

The sample size of the study was calculated with a confidence interval (CI) of 95% and an acceptable error of 2.5% on the basis of the study by Borhani Haghighi, et al.^6^ in which the knowledge of people about warning signs of stroke was assessed. We calculated that the study can be performed with 649 individuals using the following formula:

$n=\frac{Z_{1-\alpha }^{2}P(1-P)}{d^{2}}$

The questionnaire was modified to suit individual local socio-cultural condition. For assessing the content validity of questionnaire, 10 independent academic experts were invited to review questionnaire based on content validity ratio (CVR) indexes and content validity index (CVI). CVR was used for assessing the importance and accuracy of items. Based on Lawshe table, the CVR value of all items were higher than 62%. Therefore, all items considered as necessary items in the questionnaire. CVI was used for assessing congruency of each item. The CVI for each item was in range of 0.7-1.0. CVI less than 0.7 was unacceptable; 0.7-0.8 needed major revision; 0.8-0.9 needed minor revision and modification; and CVI ≥ 0.9 was acceptable without any revision. According to expert’s opinions, all questions had high CVI and CVR values for quantitative validity. Reliability of the questionnaire was also assessed using simultaneous method based on results of a pilot study (n = 25) (reliability more than 90%). The internal consistency of the questionnaire as calculated by Kuder-Richardson 20 coefficients was considered acceptable (α = 0.79). Kuder-Richardson 20 coefficients > 0.70 was considered acceptable for internal consistency.

In addition to demographic characteristics (i.e., age, gender, profession, and educational level), the questionnaire composed of 36 questions in three sections. The first section consisted of six closed-ended questions about the source of information and approach to stroke (i.e. stroke in relatives or friends, previous information about stroke, interest to have information about stroke, sources of information, recommended sources of information, and encountering patients with symptoms suggesting stroke). The second section included fifteen closed-ended questions evaluating the awareness of the participants about symptoms and warning symptoms (i.e., numbness or weakness of one side of the body, difficulty speaking or understanding speech, double or blurred vision, severe headache, and dizziness). The third section included fifteen closed-ended questions regarding the awareness of the participants about the risk factors (i.e., hypertension, hyperlipidemia, smoking, obesity, previous stroke, diabetes mellitus (DM), alcoholism, oral contraceptives, heart disease, and positive family history for stroke). The individuals’ awareness of stroke warning signs and risk factors is classified into three categories: poor (equal or fewer than 5 correct answers), moderate (6-10 correct answers), and good (more than 10 correct answers). The overall awareness level was defined as a percentage score of the number of correct answers in all sections divided by the total number of answers. 

Statistical analysis was done by SPSS for Windows (version 18, SPSS Inc., Chicago, IL, USA). Descriptive data were reported as percentages, frequencies, or mean ± standard deviation (SD). The normality of variable distribution was checked by the Kolmogorov-Smirnov test. Mann Whitney U Test was used to determine differences between mean values. Kruskal-Wallis Test was used to compare the frequencies of variables with more than two groups. Multiple linear regression model was used to examine the predictors of the overall level of stroke knowledge. Variables with a P-value ≤ 0.01 were included in the final stepwise model. P-value less than 0.05 was considered significant.

## Results

In this study, 649 subjects with the mean age of 32.0 ± 12.2 years (ranging from 15 to 80 years) were interviewed; and 75.0% of respondents were younger than 43 years old. Women constituted 56.4% of the study population; 29.6% of subjects were self-employed and 51.6% had academic education (Table 1).

**Table 1:** Demographic data of subjects (n = 649)

**Variables**	**n (%)**
Gender	
Male	283 (43.6)
Female	366 (56.4)
Occupation	
Studies	98 (15.1)
Housewife	164 (25.3)
Employee	181 (27.9)
Medical care jobs	14 (2.2)
Self-employment	192 (29.6)
Education	
College education	335 (51.6)
Diploma	179 (27.6)
Less than diploma	135 (20.8)

This study showed that 26.8% of subjects knew someone in their family who had a stroke. About 55.8% of subjects had previous information about stroke and 92.0% were interested to obtain information about stroke.

The most common sources of information in respondents were family (21.1%) and media (17.3%). Most subjects (65.5%) recommended “mass media” as the best source of information about stroke. When encountering a patient with symptoms consistent with stroke, 82.4% of subjects would notify emergency medical systems (EMS); 92.8% of them would refer the patient to a neurologist and 90.9% believed that the patient should immediately be transferred to a specialized healthcare center in less than 3 hours to receive adequate treatment. The source of information and approach of respondents about stroke is shown in table 2.

The awareness of participants toward risk factors and warning signs of stroke is shown in table 3. Hypertension (82.3%) and previous history of stroke (78.6%) were the major factors reported by participants, while oral contraceptive pill (OCP) (12.8%) and DM (38.8%) were not reported commonly. In addition, opium use (82.1%) and depression (91.0%) were the most common factors not correctly identified as major risk factors. The awareness about risk factors was poor in 48.8%, moderate in 39.9% and good in 11.3% of respondents. 

**Table 2:** The source of information and approach of respondents about stroke (n = 649)

**Variables**	**n (%)**
Stroke in relatives or friends	
Yes	174 (26.8)
No	475 (73.2)
Previous information about stroke	
Yes	362 (55.8)
No	287 (44.2)
Interest to have an information about stroke	
Yes	597 (92.0)
No	59 (8.0)
Sources of information	
Family members and friends	137 (21.1)
Television and radio	112 (17.3)
Reading book	38 (5.9)
Newspapers	15 (2.3)
Others	240 (37.0)
Multisource	107 (16.5)
Recommended sources of information	
Mass media audiovisual	425 (65.5)
Educational booklets	100 (15.4)
Others	52 (8.0)
Multisource	72 (11.1)
Encounter patients with symptoms suggesting stroke	
Telephone EMS	535 (82.4)
Refer to neurologist	602 (92.8)
Need medical help immediately (before 3 hours)	590 (90.9)

EMS: emergency medical systems

**Table 3:** Awareness of subjects about risk factors and warning symptoms

**Variables**	**Correct**	**Incorrect**	**No information**
Risk factors [n (%)]			
Hypertension	534 (82.3)	16 (2.5)	99 (15.3)
Smoking	397 (61.2)	62 (9.6)	190 (29.3)
DM	252 (38.8)	111 (17.1)	286 (44.1)
Hypercholesterolemia	288 (44.4)	361 (55.6)	-
Alcohol abuse	332 (51.2)	72 (11.1)	289 (44.5)
History of cardiac disease	260 (40.1)	128 (19.7)	261 (40.2)
Family history of stroke	477 (73.5)	46 (7.1)	126 (19.4)
Past history of stroke	510 (78.6)	25 (3.9)	114 (17.6)
Aging	482 (74.3)	39 (6.0)	128 (19.7)
OCP	83 (12.8)	184 (28.4)	382 (58.9)
Opium	116 (17.9)	207 (31.9)	326 (50.2)
Peptic ulcer	286 (44.1)	252 (38.8)	286 (44.1)
Hair coloring	20 (32.0)	57 (8.8)	384 (59.2)
Depression	56 (8.6)	328 (50.2)	265 (40.8)
Obesity	327 (50.4)	56 (8.6)	266 (41.0)
Warning symptoms [n (%)]			
Sudden weakness of a leg	279 (43.0)	88 (13.6)	282 (43.5)
Sudden weakness of an arm	268 (41.3)	88 (13.6)	293 (45.1)
Sudden pain of unilateral limbs	141 (21.7)	172 (26.5)	336 (51.8)
Sudden severe abdominal pain	290 (44.7)	44 (6.8)	315 (48.5)
Sudden ataxia and vertigo	418 (64.4)	25 (3.9)	206 (31.7)
Sudden epistaxis	270 (41.6)	99 (15.3)	280 (43.1)
Sudden difficulty to speak	407 (62.7)	41 (6.3)	201 (31.0)
Sudden difficulty to understand	374 (57.6)	44 (6.8)	231 (35.6)
Sudden perspiration	89 (13.7)	245 (37.8)	315 (48.5)
Sudden chest pain	263 (40.5)	96 (14.8)	290 (44.7)
Sudden visual defect in one eye	322 (49.6)	79 (12.2)	248 (38.2)
Sudden and severe headache	330 (50.8)	50 (7.7)	269 (41.4)
Sudden diplopia	278 (42.8)	68 (10.5)	303 (46.7)
Sudden loss of consciousness	424 (65.3)	30 (4.6)	195 (30.0)
Seizure	75 (11.6)	328 (50.5)	246 (37.9)

OCP: Oral contraceptive pill; DM: Diabetes mellitus

**Table 4:** Results of multiple linear regressions in variables related to subjects awareness

**Variables**	**β-coefficient**	**SE**	**P**	**95% CI of coefficient**	
				**Upper limit**	**Lower limit**
Age	0.27	0.07	< 0.001	0.13	0.4
Previous information about stroke	18.70	1.59	< 0.001	15.60	21.8
Students	6.41	2.49	0.010	1.51	11.3
Housewives	8.90	1.89	< 0.001	1.51	12.6
Medical care jobs	13.20	5.44	0.016	2.48	23.9

CI: Confidence interval; SE: Standard error

The loss of consciousness (65.5%), as well as vertigo and ataxia (64.4%), were reported as the most common warning signs of stroke. On the other hand, sudden perspiration (86.3%) and unilateral pain in limbs (78.3%) were the most correctly identified incorrect responses. In addition, the weakness of an arm (41.3%) and diplopia (42.8%) were less commonly identified as the warning signs of stroke. Totally, the awareness of stroke warning signs was poor in 51.8%, moderate in 34.8% and good in 13.4% of respondents.

The approach to patients with stroke was not significantly associated with age (P = 0.700), sex (P = 0.345), educational level (P = 0.084), job (P = 0.340), family history of ischemic stroke (P = 0.100), previous knowledge about stroke (P = 0.130), and the source of information (P = 0.060). However, the awareness of people regarding the risk factors of stroke was significantly related to age (P < 0.001), sex (P = 0.029), educational level (P = 0.006), job (P < 0.001), family history of ischemic stroke (P < 0.001), having previous knowledge about stroke (P < 0.001), and the source of information (P = 0.050). Moreover, the awareness of people about stroke warning signs was significantly associated with age (P < 0.001), sex (P = 0.008), educational level (P = 0.004), job (P < 0.001), family history of ischemic stroke (P < 0.001), having previous knowledge about stroke (P < 0.001), and the source of information (P = 0.018). 

Totally, the overall mean percentage score of subjects’ awareness about risk factors and the warning signs of stroke was 54.4 ± 22.8 (ranging from 3.03 to 93.9). In addition, the total awareness in 75 % of participants was more than 70%. In total, the overall awareness of people about stroke was associated with gender (P = 0.017), educational level (P = 0.016), job (P = 0.001), family history of ischemic stroke (P = 0.001), previous knowledge about stroke (P = 0.001), and the source of information (P = 0.050). Multiple linear regressions showed that age (β = 0.277, P < 0.001), the academic level of education (β = 6.41, P = 0.010), housewifery (β = 8.9, P < 0.001), jobs related to medical care and the previous information about stroke (β = 18.71, P < 0.001) were significant predictors of the overall awareness of patients about stroke (Table 4) as the overall awareness level of subjects increased by 0.27% in proportion to every year of increase in age. It also increased by 18.7% when people had previous information about stroke.

## Discussion

To propagate efficient treatment-seeking behavior and to bring the correct message appropriately, the assessment of public needs for information should precede the development and implementation of educational campaigns for the public.^9^ The early detection of stroke risk factors and warning signs has an important role in the prevention and management of patients with stroke.^6^ The lack of information about stroke can disarrange the prevention programs and delay the rapid medical intervention. This study was the first population-based telephone survey in Iran which assessed the public awareness of stroke, warning signs and risk factors in Rasht. This study has shown that the awareness about stroke, its risk factors and warning signs is adequate, and it can be related to significant factors such as education and the source of information.

Consistent with a study by Borhani Haghighi, et al.,^6^ hypertension was a major risk factor reported by our study population. In a study by Alaqeel, et al.,^23^ the awareness regarding stroke’s risk factor was found to be low and only 33 percent of participants reported hypertension as a risk factor. It seems that in Iran people are appropriately informed about hypertension and its complications. In this study, depression and a history of opium use were incorrectly considered as risk factors by considerable percentage of participants; while DM and hyperlipidemia were not identified as major risk factors. Considering the role of multimedia as one of the most important sources of information in our study, further public education using various media sources including television, radio, newspaper, magazine, and educational pamphlets is needed to improve the awareness of community regarding stroke’s risk factors. 

The loss of consciousness as well as vertigo and ataxia were the major warning signs of stroke identified by participants. However, a few number of respondents reported paresthesia and aphasia as warning signs. In addition, sudden chest pain and perspiration were reported by some respondents. In the only community-based, face-to-face interview survey conducted in Iran, Borhani Haghighi, et al. revealed that abdominal pain are one of the most commonly identified symptoms of stroke.^6^ While in another study in Korea,^24^ participants identified paresthesia as the main warning signs. Therefore, most of the educational efforts in future should be focused on increasing the awareness of Iranian community about stroke’s warning signs.

In our study, the mean percentage score of public awareness about risk factors and warning signs was 54.4% and it was more than 70.0% in 75.0% of cases. In a telephone survey by Pancioli, et al. 57% of subjects knew at least one warning sign and 68% of them named at least one risk factor.^18^ In the only community-based, face-to-face interview survey in Korea by Kim, et al.,^24^ 62.0% reported at least one stroke symptom and 56.0% reported at least one risk factor for stroke in open-ended questioning. In Saudi Arabia, Alaqeel, et al.^23^ revealed that 21.7% of the respondents correctly chose ≥ 5 risk factors and made ≤ 1 error and 18.4% of the participants were able to correctly identify ≥ 3 symptoms of the list and make ≤ 1 error. 

In another large population-based telephone survey, Sug Yoon, et al.^21^ found that 76.2% of Australian individuals could name one or more risk factors of stroke; however, just 49.8% of them could identify at least one stroke warning sign. Moreover, smoking and visual disturbance were two most common risk factors and symptoms of stroke listed by 39.4% and 24.1% of respondents, respectively. The high rate of correct answers in our study is likely to be related to use of closed-ended questions in our questionnaires, in contrast to most previous studies. Therefore, further studies will need to be performed to survey the public awareness when using open ended questionnaire.

In a study by Travis, et al. in the USA, 42.0% of persons would first call EMS if having a stroke.^25^ In our study, 82.4% of respondents would immediately call EMS, 92.8% would refer to a neurologist and 90.9% suggested receiving adequate treatment in less than three hours, when they see patients with symptoms suggesting stroke. There are several possible explanations for this relatively high percentage of correct responses, compared to similar studies. First, participation of more educated people in this study. Second, higher general medical knowledge of our population. Third, identifying loss of consciousness as the most common warning sign by participants. It is therefore likely that the fear of loss of consciousness sign alone may be related to high rate of calling EMS in our study. 

The most common sources of information in our study were friends and then multimedia; moreover, the highest awareness was seen in respondents that studied books as sources of information. Kim, et al.^24^ revealed that the major source of information about stroke was television (59%), and the most reliable sources were the respondents' physicians (55%); however, among the respondents of 20 to 39 years of age, the Internet (37%) was the second greatest source of information. Alaqeel, et al.^23^ reported that 49.9% of respondents named mass media as the source of their knowledge. In a study by Stern, et al.^26^ in the USA, 657 adults were examined for the effectiveness of the slide/audio community education program lonely or accompanied by facilitation led by a trained individual. They reported that slide/audio program is effective in increasing the knowledge of stroke risk factors, warning signs, and necessary action but facilitation did not significantly affect the short-term acquisition of information.^26^ Different findings in source of information can be related to increased number of Internet users; also it showed that multimedia programs can be effective in all developing and developed countries.

This study showed that the overall awareness of people about stroke, its risk factors and warning signs was related to age, academic level of education, job and previous information about stroke. In addition, the awareness of subjects increased 0.3% in proportion to the age increase in every year, and it increased 18.7% when people had the previous information about stroke. Travis, et al. reported that the level of knowledge was higher in females, young adults, subjects with quality education, previous history of stroke or hypertension, smokers and high-income people.^25^ Borhani Haghighi, et al. showed that the attitude and knowledge were related to age, education and income but not to gender and domicile.^6^ Stern, et al. expressed that multimedia, family and friends, health professionals and educational campaigns can successfully increase stroke awareness. However, they also showed that race or educational level could not increase the knowledge.^26^ These differences can be related to cultural and other influential factors among different nations. According to the important effect of age and educational level in this study, more educational programs, especially in school age, should be planned in this region to increase the level of awareness of students. 

As a limitation, limited-sample telephone-based survey instead of face-to-face interview was done in our study. Telephone call can affect the responses of subjects. Although interviewers were trained on how to avoid leading questions, the interviewer bias might have influenced the participant response. In addition, a number of people in this region might not have had access to telephone; thus, people with low socioeconomic status may not have been included.

## Conclusion

This study concludes that the awareness of people about stroke, its risk factors and warning signs was adequate and can be related to significant factors such as education and the source of information. So it is suggested to program public multimedia and health education in academies and colleges in future to increase the knowledge and awareness of people.

## Notes:

**How to cite this article:** Hosseininezhad M, Ebrahimi H, Seyedsaadat SM, Bakhshayesh B, Asadi M, Ghayeghran AR. Awareness toward stroke in a population-based sample of Iranian adults. Iran J Neurol 2017; 16(1): 7-14.
